# Accessing simply-substituted 4-hydroxytetrahydroisoquinolines via Pomeranz–Fritsch–Bobbitt reaction with non-activated and moderately-activated systems

**DOI:** 10.3762/bjoc.13.182

**Published:** 2017-09-06

**Authors:** Marco Mottinelli, Mathew P Leese, Barry V L Potter

**Affiliations:** 1 Wolfson Laboratory of Medicinal Chemistry, Department of Pharmacy and Pharmacology, University of Bath, Claverton Down, BA2 7AY Bath, UK; 2 Drug Discovery & Medicinal Chemistry, Department of Pharmacology, University of Oxford, Mansfield Road, Oxford, OX1 3QT, UK

**Keywords:** cyclization, Pomeranz–Fritsch, steroidomimetic, synthesis, tetrahydroisoquinoline

## Abstract

**Background:** 1,2,3,4-Tetrahydroisoquinolines (THIQs) are common motifs in alkaloids and in medicinal chemistry. Synthetic access to THIQs via the Pomeranz–Fritsch–Bobbit (PFB) methodology using mineral acids for deactivated, electron-poor aromatic systems, is scarcely represented in the literature. Here, the factors controlling the regiochemical outcome of cyclization are evaluated.

**Results:** A double reductive alkylation was telescoped into a one-pot reaction delivering good to excellent yields of desired aminoacetals for cyclization. Cyclization of activated systems proceeded smoothly under standard PFB conditions, but for non-activated systems the use of HClO_4_ alone was effective. When cyclization was possible in both *para*- and *ortho*-positions to the substituent, 7-substituted derivatives were formed with significant amounts of 5-substituted byproduct. The formation of the 4-hydroxy-THIQs vs the 4-methoxy-THIQ products could be controlled through modification of the reaction concentration. In addition, while a highly-activated system exclusively cyclized to the indole, this seems generally highly disfavored. When competition between 6- and 7-ring formation was investigated in non-activated systems, 5,7,8,13-tetrahydro-6,13-methanodibenzo[*c*,*f*]azonine was exclusively obtained. Furthermore, selective ring closure in the *para-*position could be achieved under standard PFB conditions, while a double ring closure could be obtained utilizing HClO_4_.

**Conclusion:** Reactivity differences in aminoacetal precursors can be employed to control cyclization using the PFB methodology. It is now possible to select confidently the right conditions for the synthesis of *N-*aryl-4-hydroxy-1,2,3,4-tetrahydroisoquinolines.

## Introduction

**Figure 1:**
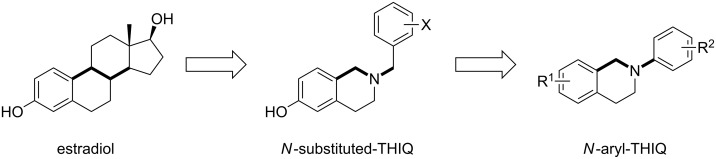
Non-steroidal templates from estradiol.

1,2,3,4-Tetrahydroisoquinoline (THIQ) motifs are present in many natural alkaloids [1]. THIQ derivatives have also been investigated as potential therapeutics in a wide range of diseases and recent studies have explored their potential as steroidomimetics [2–5]. Given the success of these authors in designing highly potent non-steroidal chimeric microtubule disruptors based upon decorated THIQ-based mimics of the steroidal AB ring system that possess pendant *N-*substituents [2], robust routes to direct *N-*aryl substituted THIQs were targeted for related activities (Figure 1).

**Scheme 1:**
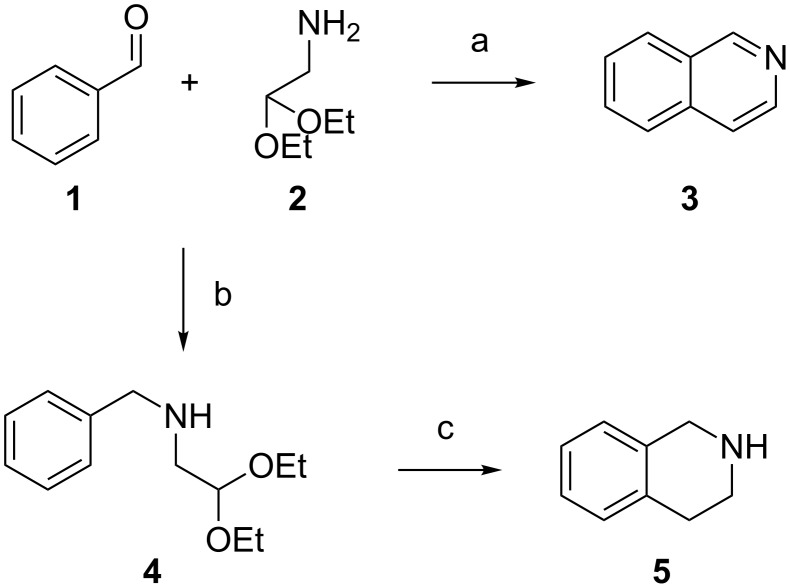
Pomeranz–Fritsch and Pomeranz–Fritsch–Bobbitt reactions. Conditions: (a) conc. H_2_SO_4_; (b) benzene, ∆, Dean–Stark then Raney Ni, H_2_, 50 psi, EtOH; (c) conc. HCl, Δ.

Isoquinolines can be obtained from benzaldehyde and 2,2-diethoxyethylamine under Pomeranz–Fritsch (PF) reaction conditions (Scheme 1), as first reported in 1893 [6–7]. A modification of the classic reaction reported by Bobbitt allows access to THIQ analogues (Scheme 1) [8–10]. Later, research has principally focused on the asymmetric THIQ synthesis and a substantial number of approaches to this end have been reported in the literature [1,11–14]. Notwithstanding this, application of the Bobbitt modification of the Pomeranz–Fritsch reaction, or Pomeranz–Fritsch–Bobbit (PFB), as an approach to simply-substituted THIQs has been somewhat neglected. In fact, most literature reports of cyclization under PFB conditions have concerned strongly-activated aromatic systems [8–12,15–18] with a few examples of cyclization of deactivated, electron-deficient, aromatic systems [19] in the presence of mineral acids. Synthetic approaches to electron poor systems more often involve different chemistries [20].

The PFB reaction proceeds via reduction of an intermediate iminoacetal to provide an aminoacetal that is then cyclized and reduced to deliver the 1,2,3,4-tetrahydroisoquinoline product. Relative to the original Pomeranz–Fritsch conditions, the Bobbitt modification features a reduced acid concentration that advantageously reduces the formation of side products [8–10].

The key cyclization in the PFB synthesis reaction is an electrophilic aromatic substitution that is strongly impacted by the effects of the substituents on the electron density of the aromatic ring in intermediate **4**.

Given our established interest in exploring structural mimetics for the steroid nucleus we were drawn to explore whether the PFB reaction could be used to access libraries of regioisomeric *N*-aryl-1,2,3,4-tetrahydroisoquinoline derivatives that might facilitate the design of new structural templates for steroid-binding receptors. We thus evaluated the robustness and flexibility of this approach to the THIQ system and considered, in particular, the factors controlling the regiochemical outcome of the reaction to direct synthetic design. Reaction conditions of the PFB methodology and opportune modifications necessary to direct such reactions towards the synthesis of the desired THIQ derivatives are reported here.

## Results and Discussion

**Scheme 2:**
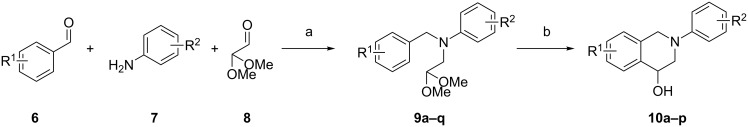
Designed synthesis of THIQ. Conditions: (a) NaBH(OAc)_3_, CHCl_3_, rt; (b) 6 M HCl or 70% HClO_4_ (see Table 1), rt.

To address aforementioned aims, we envisaged that the synthesis set out in Scheme 2 could deliver access to substrates for the PFB reaction such as **9**. After an initial, unfruitful, attempt to obtain the aminoacetals **9** from sequential condensation of aniline and alkyl and aryl halides, a double reductive alkylation that could be telescoped into a one-pot reaction was investigated. This reaction proceeded to deliver good to excellent yields of the desired aminoacetals **9a–g,i–p** accompanied by only a small amount of side products (Table 1).

**Table 1:** Double reductive alkylation.

Cmpd	R^1^	R^2^	Yield (%)	Cmpd	R^1^	R^2^	Yield (%)

**9a**	H	H	99	**9j**	4-OH	4-OMe	90
**9b**	4-OMe	H	69	**9k**	4-OH	4-Cl	66
**9c**	4-Cl	H	88	**9l**	3,4-(OMe)_2_	4-OMe	91
**9d**	4-OH	H	90	**9m**	3,4,5-(OMe)_3_	4-OMe	98
**9e**	3-Br	H	54	**9n**	2-OMe	4-Cl	96
**9f**	3-OMe	H	79	**9o**	H	3-OMe	73
**9g**	3-OMe	4-Cl	90	**9p**	H	3,4,5-(OMe)_3_	85
**9h**	4-OH	4-OH	–	**9q**	H	3,5-(OMe)_2_	–
**9i**	4-OH	4-Me	94				

**Scheme 3:**
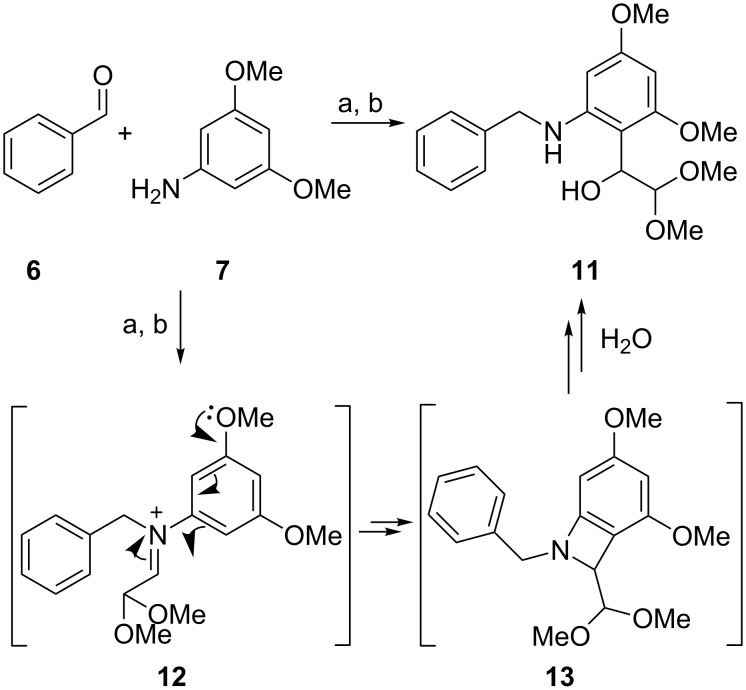
Side reaction during the double alkylation of **7** and possible reaction mechanism for the formation of **11**. Conditions: (a) NaBH(OAc)_3_, CHCl_3_, rt; (b) 2,2-dimethoxyacetaldehyde, NaBH(OAc)_3_, rt.

As an exception to the above, the acetal **9h** did not form, which could be attributed to the poor solubility of the intermediate benzylated aniline. In addition, the reaction of 3,4-dimethoxyaniline under the same conditions did not afford **9q** and instead **11** was the sole isolable reaction product (62% yield (Scheme 3)). The reaction was reproducible and proceeded even in the absence of a reducing agent (NaBH(OAc)_3_). The preferential formation of **11** relative to imine reduction, even in the presence of an excess of reducing agent, suggests a rapid intramolecular rearrangement of an iminium derivative **12** to the highly activated *ortho-*position of the aromatic ring. The possible rearrangement product **13** would then lead to the isolated compound **11** upon hydration, either during the reaction or the work-up. However, the precise mechanism for the formation of **11** has yet to be explored and efforts to unravel it lay outside the scope of this work.

The addition of 6 M HCl led to the cyclization of the 3-MeO compound **9f**, that bears an electron-donating group in a position *para*- to the cyclization point. However, the same conditions were unable to catalyze the cyclization of 4-MeO compound **9b**. We thus decided to proceed with an initial screen of acids for cyclization of non-activated compounds (Table 2).

**Table 2:** Screening of the acidic catalyst for the PFB reaction.

SM	Acid (conc.)	p*K*_a_^a^	SM^b^ (%)	P (%)

**9f**	HCl (6 M)	−8.0^c^	0	Q
**9f**	HCl (36%)	−8.0^c^	0	Q
**9f**	H_2_SO_4_ (97%)	−3.0^c^	0	0^d^
**9f**	TFA	−0.25^c^	100	0
**9f**	HClO_4_ (70%)	−10.0^c^	0	0^d^
**9b**	HCl (6 M)	−8.0^c^	0	0^e^
**9b**^f^	HCl (6 M)	−8.0^c^	0	0^g^
**9b**	HCl (36%)	−8.0^c^	0	0^g^
**9b**	H_2_SO_4_ (97%)	−3.0^c^	0	0^d^
**9b**	TFA	−0.25^c^	100	0
**9b**	HClO_4_ (70%)	−10.0^c^	0	Q

SM (starting material), P (product), Q (quantitative); ^a^p*K*_a_ refers to the acid catalyst used in the reaction; ^b^The reaction was monitored by LC–MS and the same method was used to calculate the percentage of conversion. ^c^p*K*_a_ measured in water [21]. ^d^Degraded to unknown compound. ^e^Hemiacetal formed. ^f^Reaction was performed in a microwave at 100 °C for 30 min. ^g^The was aldehyde formed.

As previously mentioned, cyclization with activated systems*,* such as the one for compound **9f**, proceeds smoothly with 6 M HCl, whereas the stronger acids H_2_SO_4_ and HClO_4_ triggered the degradation of the starting material (Table 2). In contrast, for a non-activated system, e.g., **9b**, HClO_4_ was the only acid able to deliver the desired THIQ **10b**. However, in the presence of a deactivating, *ortho/para*-directing group, such as chlorine (**9c**) or bromine (**9e**), HClO_4_ afforded the desired cyclized product only in the position directed by the substituent. In addition, even HClO_4_ failed to catalyze the cyclization of 2-MeO compound **9n**, rendering the target THIQ **10n** inaccessible by this approach.

**Scheme 4:**
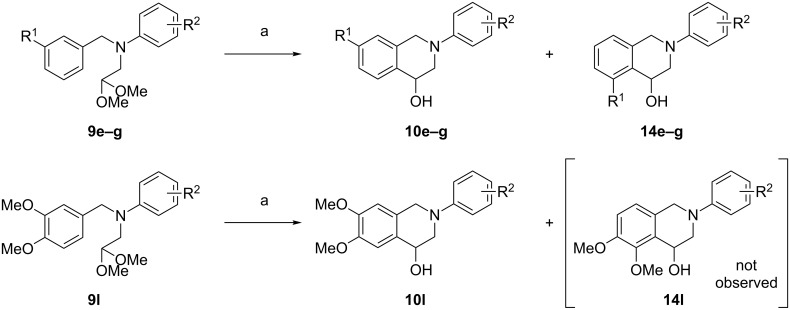
Competition between the formation of 5- and 7-substituted THIQs. Conditions: (a) 6 M HCl, rt.

In certain cases, during scale-up of the reaction to a gram scale, a minor product became clearly identifiable and could sometimes be isolated. Whenever the cyclization was possible in both the *para*- and the *ortho*-position to the substituent, as expected, the *para*-position predominated and the 7-substituted THIQs (e.g., **10e–g**, Scheme 4) were obtained as the major product. Nevertheless, it was possible to isolate a usable amount of the 5-substituted THIQ **14e**. The ratio of the two regioisomers proved to be fairly constant, ranging from 5:1 to 4:1 for the three compounds considered. However, when more than one substituent was present, i.e., **9l**, only compound **10l** formed and the alternative product **14l** was not observed.

A kinetic NMR study was performed by running a sample reaction directly in an NMR tube. However, the strongly ionic environment did not allow for a sufficient resolution of the NMR spectra and instead only extremely broad and convoluted peaks were observed. The kinetic study was then repeated by sampling the reaction at regular intervals, showing an almost instant hydrolysis of the acetal **9l** to give the corresponding aldehyde followed by its rapid conversion into THIQ **10l** within the first ten minutes of the reaction.

**Scheme 5:**
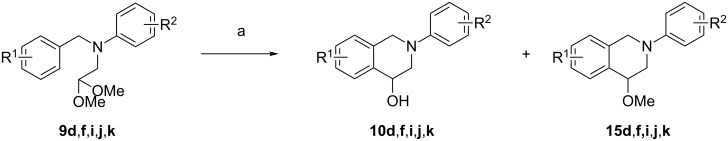
Formation of the 4-hydroxy and 4-methoxy-THIQs. Conditions: (a) 6 M HCl or 70% HClO_4_, rt (see Table 3).

Despite the indication that the reaction proceeded through an initial complete conversion of the acetal to the aldehyde, in certain cases the formation of the ethers **15** was identified (Scheme 5). The formation of the two derivatives could be controlled through changes in the reaction concentration (Table 3). When **9f** was dissolved in sufficient 6 M HCl necessary to provide a 1 M solution of the acetal, the main product obtained was the 4-hydroxy-THIQ. However, when 70% HClO_4_ was used, the ratio between the 4-hydroxy and 4-methoxy-THIQs varied from ca. 1:1 to approaching 1:0, depending on the concentration of starting material in the reaction mixture. Ultimately, it was postulated that the ratio between formation of the ether and the alcohol is most likely a function of the water content of the reaction mixture. An acetal concentration of 0.3 M proved optimal to minimize the ether formation.

**Table 3:** Ratio between the formation of **10d**,**f**,**i**,**j**,**k** and **15d**,**f**,**i**,**j**,**k** at different reaction concentrations.

SM	Conc. (M)	Products	Ratio^a^

**9d**^b^	0.9	**10d** + **15d**	2:1
**9d**^b^	0.3	**10d** + **15d**	1:0
**9f**^c^	1.0	**10f** + **15f**	5:1
**9i**^b^	0.7	**10i** + **15i**	1:1
**9j**^b^	0.8	**10j** + **15j**	1:1
**9k**^b^	0.9	**10k** + **15k**	1:1

^a^Molar ratios **10d**,**f**,**i–k** vs **15d**,**f**,**i–k** were calculated by ^1^H NMR spectroscopy of the crude reaction mixtures. ^b^Reaction was performed with 70% HClO_4_. ^c^Reaction was performed with 6 M HCl.

**Scheme 6:**
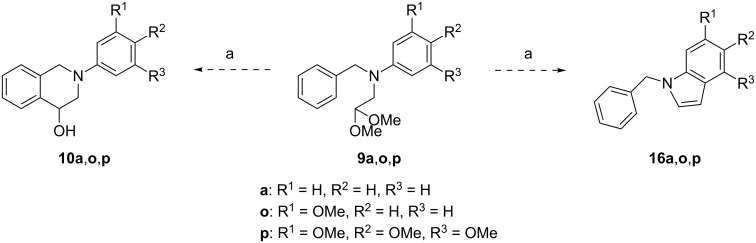
Competition between the formation of THIQs **10a**,**o**,**p** and indoles **16a**,**o**,**p**. Conditions: (a) 70% HClO_4_, rt.

The PF reaction conditions could possibly lead to a competition between 6-membered and 5-membered ring formation in systems such as **9a**,**o**,**p** (Scheme 6). For this study, compounds bearing a different number of methoxy groups were selected in order to understand the electronic constrains that might render indole formation competitive. Upon treatment with 70% HClO_4_, acetals **9a** and **9o** yielded the respective THIQs **10a** and **10o**. However, the highly-activated acetal **9p** exclusively cyclized into the indole **16p**. It is thus possible to infer that indole formation is highly disfavored and only the presence of a great number of activating groups can make this outcome competitive.

**Scheme 7:**
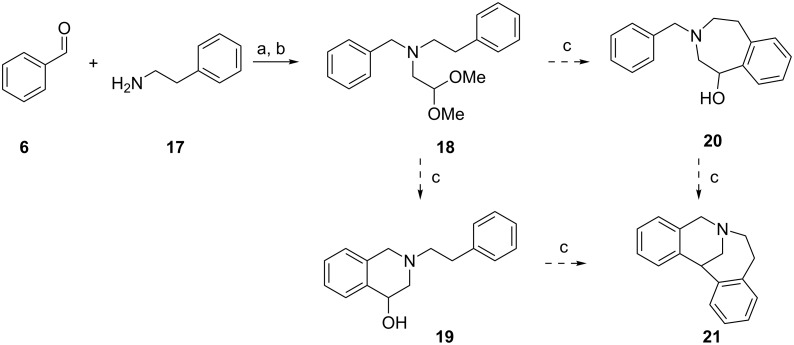
Competition between 6- and 7-membered ring formation. Conditions: (a) NaBH(OAc)_3_, CHCl_3_, rt; (b) 2,2-dimethoxyacetaldehyde, NaBH(OAc)_3_, rt; (c) 70% HClO_4_, rt.

We then decided to expand our analysis to include competition between 6- and 7-membered ring formation. Compound **18** (Scheme 7) was generated via a double reductive amination, in analogy to the syntheses of compounds **9a–q**. Because none of the aromatic rings contained any activating group, the cyclization was performed with 70% HClO_4_ as catalyst. However, in the experimental conditions used it was not possible to isolate either compound **19** or **20** (Scheme 7). Instead, the reaction proceeded to give complete conversion of **18** into the doubly-cyclized **21**. Hence, the experimental conditions used did not allow to discriminate between 6- and 7-membered ring formation.

**Scheme 8:**
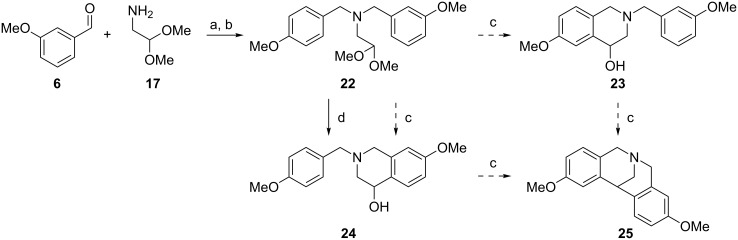
Competition between ring formation *para*- or *meta*- to an activating group. Conditions: (a) NaBH(OAc)_3_, CHCl_3_, rt; (b) 4-methoxybenzaldehyde, NaBH(OAc)_3_, rt; (c) 70% HClO_4_, rt; (d) 6 M HCl, rt.

Lastly, we investigated potential competition between ring formation in *para*- or *meta*-position to an activating group such as a methoxy group, when both were possible. For this, compound **22** (Scheme 8) was synthesized via a double reductive amination, as per synthesis of **9a–q**. As expected, when the precursor was treated with 6 M HCl, the cyclization occurred only in the *para-*position. However, when 70% HClO_4_ was used, compound **22** afforded exclusively the double ring-closed product **25** (Scheme 8), analogous to **21**. Presumably, in this case the six-membered ring *para*-cyclization may occur first followed by that directed by the six ring *meta*-position. Therefore, under our experimental conditions it was possible to obtain selectively the desired mono-cyclized product solely when this contained the activating group in the *para-*position.

## Conclusion

The classical PFB conditions could be used successfully when at least one activating group was present in a position *para* to the cyclization point. When the substitution position was not *para* or the group was not an electron-donating group, the PFB conditions were not successful and the target THIQ could be obtained only with the use of 70% HClO_4_ as a catalyst. This difference in reactivity could be exploited to control the cyclization point when both conditions were present, e.g., compound **22**. In addition, in more simply substituted substrates, e.g., **9e–g**, the formation of both 5- and 7-THIQs was observed in a somewhat constant ratio. This was not observed for compounds bearing more than one substituent. We noticed a strong preference for the 6-membered ring formation over the 5-membered ring, and the latter formed only in a greatly activated system. Conversely, no preference between 6- and 7-membered ring formation was observed. In addition, a 4-MeO-THIQ side product may be identified and its formation could be controlled by modifying the concentration of the starting material in the reaction media. In summary, the reported findings reveal the possibility to select confidently the appropriate conditions to synthesize the desired *N*-aryl-4-hydroxy-1,2,3,4-tetrahydroisoquinolines and derivatives to be explored as steroidomimetics in medicinal chemistry. Further investigation is currently being conducted and the findings will be reported upon their availability.

## Experimental

### Materials and methods

All chemicals were purchased from Aldrich Chemical Co. or Alfa Aesar. Organic solvents of A. R. grade were supplied by Fisher Scientific. Thin-layer chromatography (TLC) was performed on precoated plates (Merck TLC aluminum sheets silica gel 60 F254). Product(s) and starting material(s) were detected by either TLC and/or LC–MS. Flash column chromatography was performed on RediSep^®^ prepacked columns (normal phase and reversed phase) with an Isco CombiFlash^®^ Rf. NMR (400 MHz or 500 MHz) spectra were recorded with Bruker AMX systems and chemical shifts are reported in parts per million (ppm). HPLC and low-resolution mass spectra analyses were obtained on a Waters Micromass ZQ equipped with a Waters 996 PDA detector using either a Waters Radialpack C18 reversed-phase column (8 × 100 mm), or a Symmetry C18 reversed-phase column (4.6 × 150 mm) eluting with the solvent system specified at 1.0 mL/min. High-resolution mass spectra (HRMS) were recorded at the Mass Spectrometry Service Centre, University of Bath, on a Bruker microTOF. Melting points were determined using an Optimelt block and are uncorrected.

2,2-Dimethoxyacetaldehyde was purchased as an aqueous solution and was extracted in CHCl_3_ before use. Petroleum ether (pet. ether) used for chromatography was the 40–60 °C distillate. The general procedures were followed unless indicated otherwise. Some representative examples of spectroscopic data are reported below. The complete sets of data are reported in Supporting Information File 1.

### General method for the double reductive amination reaction

NaBH(OAc)_3_ (3.3 g, 15 mmol) was added to a stirring solution of benzaldehyde (1.0 mL, 10 mmol) and aniline (1.1 mL, 12 mmol) in CHCl_3_ (60 mL) and the mixture was stirred at room temperature (rt) for one hour (h). 2,2-Dimethoxyacetaldehyde (30 mmol) was then introduced into the reaction mixture followed by NaBH(OAc)_3_ (3.3 g, 15.0 mmol) and the resultant mixture was stirred at rt for further 8 h. The mixture was then quenched with saturated aqueous solution of K_2_CO_3_ (60 mL) and the aqueous (aq) layer was extracted with CHCl_3_ (2 × 30 mL). The combined organic layers were dried with MgSO_4_, filtered and evaporated to give the crude compound **9a** as a pale yellow oil (3.87 g).

***N*-Benzyl-*N*-(2,2-dimethoxyethyl)aniline (9a).** The crude compound was purified by column chromatography (eluent: from 0–30% EtOAc in pet. ether) to give **9a** as a colorless oil (2.92 g, 99%) which showed: ^1^H NMR (500 MHz, CDCl_3_) δ 3.41 (s, 6H, OCH_3_), 3.58 (d, *J* = 5.1 Hz, 2H, NC*H_2_*CH), 4.63 (t, *J* = 5.1 Hz, 1H, CH(OR)_2_), 4.67 (s, 2H, CH_2_Ar), 6.70 (tt, *J* = 0.9, 7.4 Hz, 1H, ArH), 6.74 (dd, *J* = 0.9, 8.9 Hz, 2H, ArH), 7.16–7.25 (5H, m, ArH), 7.27–7.34 (m, 2H, ArH) ppm; ^13^C NMR (126 MHz, CDCl_3_) δ 53.9 (*C*H_2_CH), 54.6 (CH_3_), 54.9 (ArCH_2_), 103.5 (*C*H(OR)_2_), 112.3 (ArCH), 116.7 (ArCH), 126.6 (ArCH), 126.8 (ArCH), 128.7 (ArCH), 129.4 (ArCH), 138.9 (Ar*C*CH_2_) and 148.7 (ArCN) ppm; LC–MS (ES^+^) *t*_R_ = 1.81 min (87%), *m*/*z* 226.0 (M + H)^+^; HRMS (ES^+^): (M + H)^+^ calcd. for C_17_H_22_NO_2_, 272.1645; found, 272.1651.

### General method for the PF cyclization with HClO_4_ (method A)

Compound **9a** (3.0 g, 11.1 mmol) was dissolved in 70% HClO_4_ (33 mL) and stirred for 1 h at rt. The mixture was then diluted with water (30 mL) and basified by carefully pouring the mixture over Na_2_CO_3_. The aq layer was then extracted with EtOAc (3 × 30 mL) and the combined organic layers were dried with MgSO_4_, filtered and evaporated to give a brown foam (2.97 g).

### General method for the PF cyclization with HCl (method B)

Compound **9f** (500 mg, 1.66 mmol) was dissolved in 6 M HCl (2 mL) and stirred at rt for 1 h during which time the mixture turned red. The reaction mixture was cooled to 0 °C and then quenched by the slow addition of aq 3 M NaOH (10 mL) (a white suspension with a yellow precipitate formed). The mixture was then extracted with EtOAc (3 × 20 mL). The organic layer was dried with MgSO_4_, filtered and evaporated to give a yellow-brown oil (445 mg).

**2-Phenyl-1,2,3,4-tetrahydroisoquinolin-4-ol (10a).** The compound was synthesized according to method A. A sample of the crude compound was purified by column chromatography (eluent: from 0% to 10% EtOAc in pet. ether) to give a yellow oil which showed: ^1^H NMR (500 MHz, CDCl_3_) δ 2.65 (bs, 1H, OH), 3.39 (dd, *J* = 2.6, 12.6 Hz, 1H, H_3_-THIQ), 3.86 (ddd, *J* = 1.1, 3.8, 12.6 Hz, 1H, H_3_-THIQ), 4.20 (d, *J* = 15.4 Hz, 1H, H_1_-THIQ), 4.49 (d, *J* = 15.4 Hz, 1H, H_1_-THIQ), 4.79 (bs, 1H, H_4_-THIQ), 6.94 (tt, *J* = 1.1, 7.4 Hz, 1H, ArH, phenyl), 7.09 (dd, *J* = 1.0, 8.8 Hz, 2H, ArH, phenyl), 7.17–7.23 (m, 1H), 7.29–7.32 (m, 2H, H_6_,H_7_-THIQ ), 7.34 (dd, *J* = 7.3, 8.8 Hz, 2H, ArH, phenyl), 7.47–7.51 (m, 1H) ppm; ^13^C NMR (126 MHz, CDCl_3_) δ 51.4 (C_1_-THIQ), 55.6 (C_3_-THIQ), 67.3 (C_4_-THIQ), 116.6 (ArCH, phenyl), 120.2 (ArCH, phenyl), 126.5, 127.2, 128.2, 129.3, 129.4 (ArCH, phenyl), 136.7, 134.3 and 151.1 (ArCN) ppm; LC/MS (ES^+^) *t*_R_ = 1.75 min (66 %), *m*/*z* 226.0 (M^+^ + H)^+^; (RP, isocratic, 90% MeOH); HRMS (ES^+^): (M + H)^+^ calcd. for C_15_H_16_NO, 226.1226; found, 226.1234.

## Supporting Information

File 1 Synthetic and purification methodologies and spectroscopic data.

## References

[1] Stöckigt J, Antonchick A P, Wu F, Waldmann H (2011). Angew Chem, Int Ed.

[2] Leese M P, Jourdan F, Kimberley M R, Cozier G E, Thiyagarajan N, Stengel C, Regis-Lydi S, Foster P A, Newman S P, Acharya K R (2010). Chem Commun.

[3] Leese M P, Jourdan F, Dohle W, Kimberley M R, Thomas M P, Bai R, Hamel E, Ferrandis E, Potter B V L (2012). ACS Med Chem Lett.

[4] Dohle W, Leese M P, Jourdan F L, Chapman C J, Hamel E, Ferrandis E, Potter B V L (2014). ChemMedChem.

[5] Leese M P, Jourdan F L, Major M R, Dohle W, Thomas M P, Hamel E, Ferrandis E, Mahon M F, Newman S P, Purohit A (2014). ChemMedChem.

[6] Pomeranz C (1893). Monatsh Chem.

[7] Fritsch P (1893). Ber Dtsch Chem Ges.

[8] Bobbitt J M, Moore T E (1968). J Org Chem.

[9] Bobbitt J M, Winter D P, Kiely J M (1965). J Org Chem.

[10] Bobbitt J M, McNew Kiely J, Khanna K L, Ebermann R (1965). J Org Chem.

[11] Pan L, Chen R, Ni D, Xia L, Chen X (2013). Synlett.

[12] Deng X, Liang J T, Liu J, McAllister H, Schubert C, Mani N S (2007). Org Process Res Dev.

[13] Awuah E, Capretta A (2010). J Org Chem.

[14] Chrzanowska M, Rozwadowska M D (2004). Chem Rev.

[15] Chrzanowska M, Grajewska A, Meissner Z, Rozwadowska M, Wiatrowska I (2012). Tetrahedron.

[16] Grajewska A, Rozwadowska M D (2007). Tetrahedron: Asymmetry.

[17] Głuszyńska A, Rozwadowska M D (2000). Tetrahedron: Asymmetry.

[18] Bevis M J, Forbes E J, Naik N N, Uff B C (1971). Tetrahedron.

[19] Suzuki T, Takamoto M, Okamoto T, Takayama H (1986). Chem Pharm Bull.

[20] Perchonock C D, Lantos I, Finkelstein J A, Holden K G (1980). J Org Chem.

[21] Ripin D H, Evans D A (2017). pKa's of Inorganic and Oxo-Acids.

