# Supplementary material for: Accessing simply-substituted 4-hydroxytetrahydroisoquinolines via Pomeranz–Fritsch–Bobbitt reaction with non-activated and moderately-activated systems
Source: Beilstein J Org Chem. 2017 Sep 6;13:1871–8. doi: 10.3762/bjoc.13.182 (PMC5629393; doi:10.3762/bjoc.13.182)
Supplement: File 1 — Synthetic and purification methodologies and spectroscopic data. [file Beilstein_J_Org_Chem-13-1871-s001.pdf]

**Supporting Information**
**for**

# Accessing simply-substituted 4-hydroxytetrahydroisoquinolines via Pomeranz–Fritsch–Bobbitt reaction with non-activated and moderately-activated systems

Marco Mottinelli[1,¶], Mathew P. Leese[1] and Barry V. L. Potter*[1,2]

Address: [1]Wolfson Laboratory of Medicinal Chemistry, Department of Pharmacy and Pharmacology, University of Bath, Claverton Down, BA2 7AY Bath, UK and [2]Drug Discovery & Medicinal Chemistry, Department of Pharmacology, University of Oxford, Mansfield Road, Oxford, OX1 3QT, UK

[¶]Present affiliation: Department of Medicinal Chemistry, School of Pharmacy, University of Florida, 1345 Center Drive, Gainesville, Florida, 32611, USA

Email: Barry V. L. Potter* - barry.potter@pharm.ox.ac.uk

*Corresponding author

## Synthetic and purification methodologies and spectroscopic data

**General method for the double reductive amination reaction:** $NaBH(OAc)_3$ (3.3 g, 15 mmol) was added to a stirring solution of benzaldehyde (1.0 mL, 10 mmol) and aniline (1.1 mL, 12 mmol) in $CHCl_3$ (60 mL) and the mixture was stirred at rt for one hour. 2,2-Dimethoxyacetaldehyde (30 mmol) was then introduced into the reaction mixture followed by $NaBH(OAc)_3$ (3.3 g, 15.0 mmol) and the resultant mixture was stirred at rt for further 8 h. The mixture was then quenched with saturated aqueous solution of $K_2CO_3$ (60 mL) and the aqueous layer was extracted with $CHCl_3$ (2 × 30 mL). The combined organics were dried with $MgSO_4$, filtered and evaporated to give the crude compound **9a** as a pale yellow oil (3.87 g).

***N*-(2,2-Dimethoxyethyl)-*N*-(4-methoxybenzyl)aniline (9b)**

The crude compound was purified by column chromatography (from 0% to 10% EtOAc in pet. ether) to give the product as a yellowish oil (2.09 g, 69%). $^1$H NMR (400 MHz, $CDCl_3$) δ 3.41 (6H, s, CHOC$\underline{H}_3$), 3.56 (2H, d, *J* = 5.0 Hz, NC$\underline{H}_2$CH), 3.79 (3H, s, $ArOCH_3$), 4.60 - 4.65 (3H, m, C$\underline{H}$$(OCH_3)_2$, $ArCH_2$), 6.61 - 6.73 (1H, m, ArH), 6.76 (2H, d, *J* = 8.3 Hz, ArH), 6.85 (2H, d, *J* = 8.3 Hz, ArH), 7.14 (2H, d, *J* = 8.4 Hz, ArH) and 7.20 (2H, t, *J* = 7.8 Hz, ArH) ppm. $^{13}$C NMR (101 MHz, $CDCl_3$) δ 53.7 (N$\underline{C}$$H_2$CH), 54.3 ($ArOCH_3$), 54.6 (CH(O$\underline{C}$$H_3)_2$), 55.4 (ArCH2), 103.4 ($CH(OR)_2$), 112.4 (ArCH), 114.1 (ArCH), 116.6 (ArCH), 127.8 (ArCH), 129.3 (ArCH), 130.7 ($ArCCH_2$), 148.7 (ArCN) and 158.6 (Ar$\underline{C}$$OCH_3$) ppm. LC/MS (ES+) $t_r$ = 2.46 min (70%), m/z 302.2 ($M^+$+H); HRMS (ES+) calcd. for $C_{18}H_{24}NO_3$ ($M^+$+H) 302.1751, found 302.1761.

***N*-(4-Chlorobenzyl)-*N*-(2,2-dimethoxyethyl)aniline (9c)**

The crude compound was purified by column chromatography (eluent: pet. ether) to give a colorless oil (2.73 g, 88%) which showed: $^1$H NMR (500 MHz, CDCl3) δ 3.40 (6H, s) (2 x $OCH_3$), 3.55 (2H, d, *J* = 5.1 Hz) (CHC$\underline{H}_2$), 4.61 (1H, t, *J* = 5.0 Hz) (OC$\underline{H}$), 4.62 (2H, s)

($ArCH_2N$), 6.70 (2H, d, *J* = 8.9 Hz) (2 x ArCH, aniline), 6.71 (1H, t, *J* = 7.3 Hz) (ArCH, aniline), 7.14 (2H, d, *J* = 8.6 Hz) (2 x ArCH, benzyl), 7.19 (2H, dd, *J* = 7.3, 8.9 Hz) (2 x ArCH, aniline) and 7.26 (2H, d, *J* = 8.6 Hz) (2 x ArCH, benzyl) ppm. $^{13}C$ NMR (126 MHz, $CDCl_3$) $\delta$ 53.9 ($CH\underline{C}H_2$), 54.5 ($ArCH_2$), 54.7 (2 x $OCH_3$), 103.4 (OCH), 112.4 (2 x ArCH, aniline), 117.0 (ArCH, aniline), 128.0 (ArCH, benzyl), 128.8 (ArCH, benzyl), 129.5 (ArCH, aniline), 132.5 (ArCCl), 137.5 ($ArCCH_2$) and 148.4 (ArCN) ppm. LC/MS ($ES^+$) $t_r$ = 3.18 min (97 %), m/z 306.2 ($M^+$); (RP, Isocratic, 90% MeOH). HRMS (ES+) calcd. for $C_{17}H_{20}{}^{35}ClNO_2$ ($M^+$+H) 306.1255, found 306.1245; calcd. for $C_{17}H_{20}{}^{37}ClNO_2$ ($M^+$+H) 308.1226, found 308.1245.

**4-(((2,2-Dimethoxyethyl)(phenyl)amino)methyl)phenol (9d)**

The crude compound was purified by column chromatography (eluent: from 0% to 30% of EtOAc in pet. ether) to give the product as a colorless oil (2.5 g, 90%) which showed: $^1H$ NMR (500 MHz, $CDCl_3$) $\delta$ 3.40 (6H, s, $CH_3$), 3.54 (2H, d, *J* = 5.1 Hz, $C\underline{H_2}CH$), 4.58 (2H, s, $ArCH_2$), 4.61 (1H, t, *J* = 5.1 Hz, $CH(OR)_2$), 5.01 (1H, bs, OH), 6.70 (1H, t, *J* = 7.2 Hz, ArH), 6.74 (2H, d, *J* = 8.7 Hz, ArH) 6.74 (2H, d, *J* = 8.6 Hz, ArH), 7.06 (2H, d, *J* = 8.6 Hz, ArH) and 7.19 (2H, dd, *J* = 7.2, 8.7 Hz, ArH) ppm. $^{13}C$ NMR (126 MHz, $CDCl_3$) $\delta$ 53.6 ($\underline{C}H_2CH$), 54.3 ($ArCH_2$), 54.7 ($CH_3$), 103.5 ($CH(OR)_2$), 112.4 (ArCH), 115.5 (ArCH), 116.7 (ArCH), 128.0 (ArCH), 129.4 ($ArCCH_2$), 148.7 (ArCN) and 154.5 (ArCOH) ppm. LC/MS ($ES^+$) $t_r$ = 2.91 min (65 %), m/z 287.5 ($M^+$); (RP, Isocratic, 80% MeOH). HRMS (ES+) calcd. for $C_{17}H_{22}NO_3$ ($M^+$+H) 288.1600, found 288.1595; calcd. for $C_{17}H_{21}NNaO_3$ ($M^+$+Na) 310.1419, found 310.1421.

***N*-(3-Bromobenzyl)-*N*-(2,2-dimethoxyethyl)aniline (9e)**

The crude compound was purified by column chromatography (eluent: 0% to 10% EtOAc in pet. ether) to give the product as a colorless oil (1.9 g, 54%) which showed: $^1H$ NMR

(400 MHz, $CDCl_3$) δ 3.48 (6H, s, CHOC$\underline{H}_3$), 3.65 (2H, d, $J$ = 5.1 Hz, NC$\underline{H}_2$CH), 3.84 (3H, s, $ArOCH_3$), 4.66 - 4.75 (3H, m, $ArCH_2$, $CH(OR)_2$), 6.67 - 6.92 (6H, m, ArH) and 7.21 - 7.35 (3H, m, ArH) ppm. $^{13}$C NMR (101 MHz, $CDCl_3$) δ 53.9 (N$\underline{C}$$H_2$CH), 54.6 (CH(O$\underline{C}$$H_3$)$_2$), 54.9 ($ArOCH_3$), 55.3 ($ArCH_2N$), 103.4 ($CH(OR)_2$), 112.0 (ArCH), 112.3 (ArCH), 112.4 (ArCH), 116.7 (ArCH), 118.9 (ArCH), 129.3 (ArCH), 129.7 (ArCH), 140.8 (Ar$\underline{C}$$CH_2$), 148.6 (ArCN) and 160.0 ($ArCOCH_3$) ppm. LC/MS ($ES^+$) $t_r$ = 2.51 min (98 %), m/z 302.2 ($M^+$+H); (RP, Isocratic, 90% MeOH). HRMS (ES+) calcd. for $C_{18}H_{24}NO_3$ ($M^+$+H) 302.1751, found 302.1739.

**_N_-(2,2-Dimethoxyethyl)-_N_-(3-methoxybenzyl)aniline (9f)**

The crude product was purified by column chromatography (from 0% to 10% EtOAc in pet. ether 40-60 °C) to give the product as a pale yellow oil (3.56 g, 79 %) which showed: $^1$H NMR (400 MHz, $CDCl_3$) δ 3.48 (6H, s, CHOC$\underline{H}_3$), 3.65 (2H, d, $J$ = 5.1 Hz, NC$\underline{H}_2$CH), 3.84 (3H, s, $ArOCH_3$), 4.66 - 4.75 (3H, m, $ArCH_2$, $CH(OR)_2$), 6.67 - 6.92 (6H, m, ArH) and 7.21 - 7.35 (3H, m, ArH) ppm. $^{13}$C NMR (101 MHz, $CDCl_3$) δ 53.86 (N$\underline{C}$$H_2$CH), 54.60 (CH(O$\underline{C}$$H_3$)$_2$), 54.93 ($ArOCH_3$), 55.27 ($ArCH_2N$), 103.38 ($CH(OR)_2$), 112.0 (ArCH), 112.3 (ArCH), 112.4 (ArCH), 116.7 (ArCH), 118.9 (ArCH), 129.3 (ArCH), 129.7 (ArCH), 140.8 (Ar$\underline{C}$$CH_2$), 148.6 (ArCN) and 160.0 ($ArCOCH_3$) ppm. LC/MS ($ES^+$) $t_r$ = 2.51 min (98 %), m/z 302.2 ($M^+$+H); (RP, Isocratic, 90% MeOH). HRMS (ES+) calcd. for $C_{18}H_{24}NO_3$ ($M^+$+H) 302.1751, found 302.1739.

**4-Chloro-_N_-(2,2-dimethoxyethyl)-_N_-(3-methoxybenzyl)aniline (9g)**

The crude compound was purified by column chromatography (eluent: from 0% to 20% EtOAc in pet. ether) to give the product as a pale yellow oil (9.08 g, 90%) which showed: $^1$H NMR (500 MHz, $CDCl_3$) δ 3.40 (6H, s, CH(OC$\underline{H}_3$)$_2$), 3.54 (2H, d, $J$ = 5.1 Hz, CHC$\underline{H}_2$), 3.76 (3H, s, $ArOCH_3$), 4.58 (1H, t, $J$ = 5.1 Hz, C$\underline{H}$$CH_2$), 4.60 (2H, s, $ArCH_2N$), 6.63 (2H, d,

*J* = 9.2 Hz, ArH, aniline), 6.71 – 6.74 (1H, m, ArH, benzyl), 6.74 – 6.80 (2H, m, ArH, benzyl), 7.10 (2H, d, *J* = 9.2 Hz, ArH, aniline) and 7.22 (1H, t, *J* = 7.9 Hz, ArH, benzyl) ppm. $^{13}$C NMR (126 MHz, $CDCl_3$) δ 54.2 (CH<u>C</u>$H_2$), 54.7 (CH(O<u>C</u>$H_3)_2$), 55.1 ($ArCH_2N$), 55.3($ArOCH_3$), 103.3 (<u>C</u>H($OCH_3)_2$), 112.0 (ArCH, benzyl), 112.4 (ArCH, benzyl), 113.6 (ArCH, aniline), 118.8 (ArCH, benzyl), 121.6 (ArCCl), 129.1 (ArCH, aniline), 129.8 (ArCH, benzyl), 140.2 ($ArCCH_2$), 147.2 (ArCN) and 160.1 (ArCO) ppm. LC/MS ($ES^+$) $t_r$ = 2.91 min (98 %), m/z 336.0 ($M^+$+H); (RP, Isocratic, 90% MeOH). HRMS ($ES^+$) calcd. $C_{18}H_{23}{}^{35}ClNO_3$ ($M^+$+H) 336.1361, found 336.1353; calcd. $C_{18}H_{23}{}^{37}ClNO_3$ ($M^+$+H) 338.1331, found 338.1353

**4-(((2,2-Dimethoxyethyl)(*p*-tolyl)amino)methyl)phenol (9i)**

The crude compound was purified by column chromatography (eluent: from 0% to 30% EtOAc in pet. ether) to give the product as a colorless oil (2.83 g, 94%) which showed: $^1$H NMR (500 MHz, $CDCl_3$) δ 2.24 (3H, s, ArC<u>H</u>$_3$), 3.40 (6H, s, CH(OC<u>H</u>$_3)_2$), 3.51 (2H, d, *J* = 5.1 Hz, C<u>H</u>$_2$CH), 4.54 (2H, s, $ArCH_2$), 4.59 (1H, t, *J* = 5.1 Hz, $CH(OR)_2$), 5.10 (1H, bs, OH), 6.66 (2H, d, *J* = 8.6 Hz, ArH), 6.74 (2H, d, *J* = 8.7 Hz, ArH), 7.00 (2H, d, *J* = 8.6 Hz, ArH) and 7.06 (2H, d, *J* = 8.7 Hz, ArH) ppm. $^{13}$C NMR (126 MHz, $CDCl_3$) δ 20.1 ($ArCH_3$), 53.7 (<u>C</u>$H_2$CH), 54.4 ($ArCH_2$), 54.5 (CH(O<u>C</u>$H_3)_2$), 103.4 (<u>C</u>$H(OR)_2$), 112.5 (ArCH), 115.3 (ArCH), 125.7 (Ar<u>C</u>$CH_3$), 127.9 (ArCH), 129.7 (ArCH), 130.8 (Ar<u>C</u>$CH_2$), 146.5 (ArCN) and 154.4 (ArCOH) ppm. LC/MS ($ES^+$) $t_r$ = 1.92 min (>99 %), m/z 301.5 ($M^+$); (RP, Isocratic, 90% MeOH). HRMS (ES+) calcd. for $C_{18}H_{24}NO_3$ ($M^+$+H) 302.1751, found 302.1757.

**4-(((2,2-dimethoxyethyl)(4-methoxyphenyl)amino)methyl)phenol (9j)**

The crude compound was purified by column chromatography (eluent 0% to 40% EtOAc in pet. ether) to give the product as a colorless oil (2.87 g, 90%) which showed: $^1$H NMR (500 MHz, $CDCl_3$) δ 3.38 (6H, s, CH(OC<u>H</u>$_3)_2$), 3.46 (2H, d, *J* = 5.1 Hz, C<u>H</u>$_2$CH)), 3.74 (3H, s,

ArOC<u>H</u>$_3$), 4.48 (2H, s, ArCH$_2$), 4.55 (1H, t, $J$ = 5.1 Hz, CH(OR)$_2$), 5.12 (1H, bs, OH), 6.71 (2H, d, $J$ = 9.1 Hz, ArH), 6.74 (2H, d, $J$ = 8.6 Hz, ArH), 6.79 (2H, d, $J$ = 9.1 Hz, ArH) and 7.07 (2H, d, $J$ = 8.6 Hz, ArH) ppm. $^{13}$C NMR (126 MHz, CDCl$_3$) $\delta$ 54.4 (<u>C</u>H$_2$CH), 54.5 (CH(O<u>C</u>H$_3$)$_2$), 55.3 (ArCH$_2$), 55.9 (ArOCH$_3$), 103.6 (CH(OR)$_2$), 114.6 (ArCH), 114.9 (ArCH), 115.4 (ArCH), 128.3 (ArCH), 131.0 (Ar<u>C</u>CH$_2$), 143.5 (ArCN), 151.7 (Ar<u>C</u>OCH$_3$) and 154.6 (ArCOH) ppm. LC/MS (ES$^+$) $t_r$ = 1.56 min (97 %), m/z 317.5 (M$^+$); (RP, Isocratic, 90% MeOH). HRMS (ES+) calcd. for $C_{18}H_{24}NO_4$ (M$^+$+H) 318.1700, found 318.1698.

**4-(((4-Chlorophenyl)(2,2-dimethoxyethyl)amino)methyl)phenol (9k)**

The crude compound was purified by column chromatography (eluent: from 0% to 30% EtOAc in pet. ether) to give the product as a colorless oil (2.12 g, 66%) which showed: $^1$H NMR (500 MHz, CDCl$_3$) $\delta$ 3.39 (6H, s, CH$_3$), 3.51 (2H, d, $J$ = 5.1 Hz, C<u>H</u>$_2$CH), 4.54 (2H, s, ArCH$_2$), 4.56 (1H, t, $J$ = 5.1 Hz, CH(OR)$_2$), 5.02 (1H, bs, OH), 6.64 (2H, d, $J$ = 9.2 Hz, ArH), 6.76 (2H, d, $J$ = 8.6 Hz, ArH), 7.03 (2H, d, $J$ = 8.6 Hz, ArH) and 7.10 (2H, d, $J$ = 9.1 Hz, ArH) ppm. $^{13}$C NMR (126 MHz, CDCl$_3$) $\delta$ 53.8 (<u>C</u>H$_2$CH), 54.4 (ArCH$_2$), 54.6 (CH$_3$), 103.2 (CH(OR)$_2$), 113.6 (ArCH), 115.4 (ArCH), 121.4 (ArCCl), 127.8 (ArCH), 128.9 (ArCH), 129.1 (Ar<u>C</u>CH$_2$), 147.2 (ArCN) and 154.5 (ArCOH) ppm. LC/MS (ES$^+$) $t_r$ = 4.19 min (74 %), m/z 321.6 (M$^+$); (RP, Isocratic, 80% MeOH). HRMS (ES+) calcd. for $C_{17}H_{21}ClNO_3$ (M$^+$+H) 322.1210, found 322.1201; calcd. for $C_{17}H_{20}ClNNaO_3$ (M$^+$+Na) 344.1029, found 344.1043.

***N*-(3,4-Dimethoxybenzyl)-*N*-(2,2-dimethoxyethyl)-4-methoxyaniline (9l)**

The crude compound was purified by column chromatography (eluent: from 0% to 40% EtOAc in pet. ether) to give the product as an orange oil (6.58 g, 91%) which showed: $^1$H NMR (400 MHz, CDCl$_3$) $\delta$ 3.37 (6H, s), 3.45 (2H, t, $J$ = 4.7 Hz), 3.73 (3H, s), 3.81 (3H, s),

3.84 (3H, s), 4.48 (2H, s), 4.56 (1H, bs) and 6.69 – 6.82 (7H, m) ppm. HRMS ($ES^+$) calcd. $C_{20}H_{28}NO_5$ ($M^+$+H) 362.1962, found 362.1976.

**_N_-(2,2-Dimethoxyethyl)-4-methoxy-_N_-(3,4,5-trimethoxybenzyl)aniline (9m)**

The crude compound was purified by column chromatography (eluent: from 0% to 50% EtOAc in pet. ether) to give the product as an orange oil (7.71 g, 98%) which showed: $^1H$ NMR (400 MHz, $CDCl_3$) $\delta$ 3.37 (6H, s), 3.47 (2H, d, $J$ = 5.1 Hz), 3.74 (3H, s), 3.78 (6H, s), 3.81 (3H, s), 4.47 (2H, s), 4.56 (1H, t, $J$ = 5.1 Hz), 6.46 (2H, s), 6.72 (2H, d, $J$ = 9.2 Hz) and 6.79 (2H, d, $J$ = 9.2 Hz) ppm. HRMS ($ES^+$) calcd. $C_{21}H_{30}NO_6$ ($M^+$+H) 392.2068, found 392.2081.

**4-Chloro-_N_-(2,2-dimethoxyethyl)-_N_-(2-methoxybenzyl)aniline (9n)**

The crude compound was purified by column chromatography (from 0% to 30% EtOAc in pet. ether) to give the product as a yellow oil (4.84 g, 96%) which showed: $^1H$ NMR (500 MHz, $CDCl_3$) $\delta$ 3.40 (6H, s, $CH(OC\underline{H}_3)_2$), 3.54 (2H, d, $J$ = 5.1 Hz, $NC\underline{H}_2CH$), 3.86 (3H, s, $ArOCH_3$), 4.59 (2H, s, $ArCH_2N$), 4.62 (1H, t, $J$ = 5.1 Hz, $C\underline{H}(OCH_3)_2$), 6.66 (2H, d, $J$ = 9.1 Hz, ArCH, aniline), 6.84 (1H, td, $J$ = 1.3, 7.2 Hz, ArCH, benzyl), 6.88 (1H, d, $J$ = 8.2 Hz, ArCH, benzyl), 6.98 (1H, d, $J$ = 7.2 Hz, ArCH, benzyl), 7.10 (2H, d, $J$ = 9.1 Hz, ArCH, aniline) and 7.22 (1H, td, $J$ = 1.3, 8.2 Hz, ArCH, benzyl) ppm. $^{13}C$ NMR (126 MHz, $CDCl_3$) $\delta$ 50.6 ($ArCH_2$), 54.2 ($N\underline{C}H_2CH$), 54.7 ($CH(O\underline{C}H_3)_2$), 55.1 ($ArOCH_3$), 103.1 ($\underline{C}H(OCH_3)_2$), 110.0 (ArCH, benzyl), 113.7 (2 x ArCH, aniline), 120.3 (ArCH, benzyl), 121.8 (ArCCl), 124.9 ($Ar\underline{C}CH_2$), 127.2 (ArCH, benzyl), 127.9 (ArCH, benzyl), 128.9 (2 x ArCH, aniline), 146.7 (ArCN) and 157.1 (ArCO) ppm. HRMS ($ES^+$) calc. for $C_{18}H_{23}{}^{35}ClNO_3$ ($M^+$+H) 336.1361, found 336.1355. Calc. for $C_{18}H_{23}{}^{37}ClNO_3$ ($M^+$+H) 338.1331, found 338.1306.

**_N_-Benzyl-_N_-(2,2-dimethoxyethyl)-3-methoxyaniline (9o)**

The crude compound was purified by column chromatography (eluent: from 0% to 10% EtOAc in pet. ether) to give a colorless oil (2.21 g, 73%) which showed: $^{1}$H NMR (500 MHz, $CDCl_3$) δ 3.40 (6H, s, $CH(OC\underline{H}_3)_2$), 3.55 (2H, d, *J* = 5.1 Hz, $C\underline{H}_2CH$), 3.74 (3H, s, $ArOC\underline{H}_3$), 4.62 (1H, t, *J* = 5.1 Hz, $CH(OR)_2$), 4.65 (2H, s, $ArCH_2$), 6.28 (1H, ddd, *J* = 0.6, 2.5, 8.2 Hz, ArH), 6.30 (1H, t, *J* = 2.5 Hz, ArH), 6.36 (1H, ddd, *J* = 0.6, 2.5, 8.2 Hz, ArH), 7.10 (1H, t, *J* = 8.2 Hz, ArH), 7.19 - 7.23 (3H, m, ArH) and 7.27 - 7.32 (2H, m, ArH) ppm. $^{13}$C NMR (126 MHz, $CDCl_3$) δ 53.9 ($\underline{C}H_2CH$), 54.7 ($CH(O\underline{C}H_3)_2$), 55.0 ($ArCH_2$), 55.2 ($ArOCH_3$), 99.0 (ArCH), 101.5 (ArCH), 103.5 ($CH(OR)_2$), 105.5 (ArCH), 126.6 (ArCH), 126.8 (ArCH), 128.7 (ArCH), 130.1 (ArCH), 138.8 ($ArCCH_2$), 150.2 (ArCN) and 160.9 ($Ar\underline{C}OCH_3$) ppm. LC/MS ($ES^+$) $t_r$ = 2.48 min (96 %), m/z 301.5 ($M^+$); (RP, Isocratic, 90% MeOH). HRMS (ES+) calcd. for $C_{18}H_{24}NO_3$ ($M^+$+H) 302.1751, found 302.1738.

**_N_-Benzyl-_N_-(2,2-dimethoxyethyl)-3,4,5-trimethoxyaniline (9p)**

The crude compound was purified by column chromatography (eluent 0% to 30% EtOAc in pet. ether) to give the product as a colorless oil (3.08 g, 85%) which showed: $^{1}$H NMR (500 MHz, $CDCl_3$) δ 3.41 (6H, s, $CH(OC\underline{H}_3)_2$), 3.54 (2H, d, *J* = 5.0 Hz, $C\underline{H}_2CH$), 3.74 (6H, s, $ArOC\underline{H}_3$), 3.76 (3H, s, $ArOC\underline{H}_3$), 4.59 (1H, t, *J* = 5.0 Hz, $CH(OR)_2$), 4.60 (2H, s, $ArCH_2$), 5.97 (2H, s, ArH), 7.21 - 7.24 (3H, m, ArH) and 7.28 - 7.33 (2H, m, ArH) ppm. $^{13}$C NMR (126 MHz, $CDCl_3$) δ 54.66 ($CH(O\underline{C}H_3)_2$), 54.69 ($\underline{C}H_2CH$), 55.73 ($ArCH_2$), 56.07 ($ArOCH_3$), 61.21 ($ArOCH_3$), 91.0 (ArCH), 103.8 ($CH(OR)_2$), 126.8 (ArCH), 127.0 (ArCH), 128.7 (ArCH), 129.9 ($Ar\underline{C}OCH_3$), 139.1 ($Ar\underline{C}CH_2$), 145.8 (ArCN) and 153.8 ($Ar\underline{C}OCH_3$) ppm. LC/MS ($ES^+$) $t_r$ = 1.93 min (89 %), m/z 361.3 ($M^+$); (RP, Isocratic, 90% MeOH). HRMS (ES+) calcd. for $C_{20}H_{28}NO_5$ ($M^+$+H) 362.1962, found 362.1948.

**General method for the PF cyclization with $HClO_4$ (method A):** Compound **9a** (3.0 g, 11.1 mmol) was dissolved in 70% $HClO_4$ (33 mL) and stirred for 1 h at rt. The mixture was then diluted with water (30 mL) and basified by carefully pouring the mixture over $Na_2CO_3$. The aqueous layer was then extracted with EtOAc (3 × 30 mL) and the combined organics were dried with $MgSO_4$, filtered and evaporated to give a brown foam (2.97 g).

**General method for the PF cyclization with HCl (method B):** Compound **9f** (500 mg, 1.66 mmol) was dissolved in 6 M HCl (2 mL) and stirred at rt for 1 h during which time the mixture turned red. The reaction mixture was cooled to 0 °C and then quenched by the slow addition of aq 3 M NaOH (10 mL) (a white suspension with a yellow precipitate formed). The mixture was then extracted with EtOAc (3 × 20 mL). The organic layer was dried with $MgSO_4$, filtered and evaporated to give a yellow-brown oil (445 mg).

**2-Phenyl-1,2,3,4-tetrahydroisoquinolin-4-ol (10a)**

The compound was synthesized according to method A. A sample of crude compound was purified by column chromatography (eluent: from 0% to 10% EtOAc in pet. ether) to give a yellow oil which showed: $^{1}$H NMR (500 MHz, $CDCl_3$) $\delta$ 2.65 (1H, bs, OH), 3.39 (1H, dd, $J$ = 2.6, 12.6 Hz, $H_3$-THIQ), 3.86 (1H, ddd, $J$ = 1.1, 3.8, 12.6 Hz, $H_3$-THIQ), 4.20 (1H, d, $J$ = 15.4 Hz, $H_1$-THIQ), 4.49 (1H, d, $J$ = 15.4 Hz, $H_1$-THIQ), 4.79 (1H, bs, $H_4$-THIQ), 6.94 (1H, tt, $J$ = 1.1, 7.4 Hz, ArH, phenyl), 7.09 (2H, dd, $J$ = 1.0, 8.8 Hz, ArH, phenyl), 7.17 – 7.23 (1H, m), 7.29 – 7.32 (2H, m, $H_6$,$H_7$-THIQ ), 7.34 (2H, dd, $J$ = 7.3, 8.8 Hz, ArH, phenyl) and 7.47 – 7.51 (1H, m) ppm. $^{13}$C NMR (126 MHz, $CDCl_3$) $\delta$ 51.4 ($C_1$-THIQ), 55.6 ($C_3$-THIQ), 67.3 ($C_4$-THIQ), 116.6 (ArCH, phenyl), 120.2 (ArCH, phenyl), 126.5, 127.2, 128.2, 129.3, 129.4 (ArCH, phenyl), 136.7, 134.3 and 151.1 (ArCN) ppm. LC/MS ($ES^+$) $t_r$ = 1.75 min (66 %), m/z 226.0 ($M^+$+H); (RP, Isocratic, 90% MeOH). HRMS ($ES^+$) calcd. for $C_{15}H_{16}NO$ ($M^+$+H) 226.1226, found 226.1234.

**6-Methoxy-2-phenyl-1,2,3,4-tetrahydroisoquinolin-4-ol (10b)**

The compound was synthesized according to method A. A sample of crude compound was purified by column chromatography (eluent: from 0% to 30% EtOAc in pet. ether) to give a yellow oil which showed: $^1$H NMR (500 MHz, $CDCl_3$) δ 2.54 (1H, bs, OH), 3.37 (1H, dd, *J* = 2.6, 12.6 Hz, $H_3$-THIQ), 3.83 (3H, s, $ArOCH_3$), 3.84 (4H, ddd, *J* = 1.1, 3.8, 12.6 Hz, $H_3$-THIQ), 4.14 (1H, d, *J* = 14.9 Hz, $H_1$-THIQ), 4.43 (1H, d, *J* = 14.9 Hz, $H_1$-THIQ), 4.74 (1H, bs, $H_4$-THIQ), 6.88 (1H, dd, *J* = 2.7, 8.4 Hz, $H_7$-THIQ), 6.91 (1H, tt, *J* = 0.8, 7.4 Hz, ArH, phenyl), 7.01 (1H, d, *J* = 2.7 Hz, $H_5$-THIQ), 7.07 (2H, dd, *J* = 0.8, 8.7 Hz, ArH, phenyl), 7.10 (1H, d, *J* = 8.4 Hz, $H_8$-THIQ) and 7.32 (2H, dd, *J* = 7.3, 8.7 Hz, ArH, phenyl) ppm. $^{13}$C NMR (126 MHz, $CDCl_3$) δ 50.9 ($C_1$-THIQ), 55.5 ($ArOCH_3$), 55.5 ($C_3$-THIQ), 67.6 ($C_4$-THIQ), 113.1 ($C_5$-THIQ), 115.3 ($C_7$-THIQ), 116.6 (ArCH, phenyl), 120.2 (ArCH, phenyl), 126.4 ($C_1\underline{C}C_8$-THIQ), 127.6 ($C_8$-THIQ), 129.4 (ArCH, phenyl), 137.8 ($C_4\underline{C}C_5$-THIQ), 151.2 (ArCN) and 158.7 ($C_6$-THIQ) ppm. LC/MS (ES+) $t_r$ = 1.77 min (56%), m/z 255.9 ($M^+$+H). HRMS (ES+) calcd. for $C_{16}H_{18}NO_2$ ($M^+$+H) 256.1332, found 256.1326.

**2-Phenyl-1,2,3,4-tetrahydroisoquinoline-4,6-diol (10d)**

The compound was synthesized according to method A. The crude compound was obtained as a brown-yellow solid (2.15 g) which showed: $^1$H NMR (400 MHz, $CDCl_3$) δ 3.42 (1H, ddd, *J* = 0.6, 2.9, 12.7 Hz), 3.85 (1H, ddd, *J* = 1.0, 4.0, 12.7 Hz), 4.17 (1H, d, *J* = 14.3 Hz), 4.46 (1H, d, *J* = 14.2 Hz), 4.76 (1H, s), 6.84 (1H, dd, *J* = 2.7, 8.4 Hz), 6.92 - 7.05 (2H, m), 7.08 - 7.13 (3H, m) and 7.31 - 7.39 (3H, m) ppm. LC/MS ($ES^+$) $t_r$ = 1.22 min (81 %), m/z 242.1 ($M^+$+H); (RP, Isocratic, 90% MeOH).

**7-Bromo-2-phenyl-1,2,3,4-tetrahydroisoquinolin-4-ol (10e)**

The compound was synthesized according to method A. The crude compound was obtained as a pale yellow oil (250 mg, 48%) which showed: $^1$H NMR (500 MHz, $CDCl_3$) δ

3.19 (1H, dd, $J$ = 2.1, 12.9 Hz, C$\underline{H}_2$CH), 4.07 (1H, ddd, $J$ = 1.6, 2.4, 12.9 Hz, C$\underline{H}_2$CH), 4.11 (1H, d, $J$ = 15.2 Hz, ArCH$_2$), 4.54 (1H, d, $J$ = 15.5 Hz, ArCH$_2$), 5.03 (1H, t, $J$ = 2.3 Hz, C$\underline{H}$OH), 6.95 (1H, tt, $J$ = 0.9, 7.4 Hz, ArH), 7.10 (2H, dd, $J$ = 0.9, 8.7 Hz, ArH), 7.16 (1H, d, $J$ = 2.0 Hz, ArH), 7.17 (1H, d, $J$ = 7.2 Hz, ArH), 7.34 (2H, dd, $J$ = 7.4, 8.7 Hz, ArH) and 7.52 (1H, dd, $J$ = 2.0, 7.2 Hz, ArH) ppm. $^{13}$C NMR (126 MHz, CDCl$_3$) δ 51.5 (ArCH$_2$), 55.7 ($\underline{C}$H$_2$CH), 66.7 (CHOH), 116.8 (ArCH), 117.0 (ArCH), 120.8 (ArCH), 125.8 (ArCBr), 126.0 (ArCH), 129.4 (ArCH), 131.4 (ArCH), 135.6 (Ar$\underline{C}$CH), 137.1 (Ar$\underline{C}$CH$_2$) and 150.9 (ArCN) ppm. LC/MS (ES$^+$) $t_r$ = 1.97 min (61 %), m/z 303.4 (M$^+$+H) ($^{79}$Br), 305.4 (M$^+$+H) ($^{81}$Br); (RP, Isocratic, 90% MeOH). HRMS (ES$^+$) calcd. for $C_{15}H_{15}BrNO$ (M$^+$+H) ($^{79}$Br) 304.0332, found 304.0335; calcd. for $C_{15}H_{15}BrNO$ (M$^+$+H) ($^{81}$Br) 306.0311, found 306.0323.

**7-Methoxy-2-phenyl-1,2,3,4-tetrahydroisoquinolin-4-ol (10f)**

The compound was synthesized according to method A. The crude compound was purified with chromatography (eluent: from 0% to 25% EtOAc in pet. ether) to give the product as a yellow oil (302 mg, 71%) which showed: $^1$H NMR (400 MHz, CDCl$_3$) $\delta$ 2.54 (1H, bs), 3.33 (2H, dd, $J$ = 2.3, 12.6 Hz), 3.81 (4H, s), 3.87 (2H, dd, $J$ = 3.0, 12.6 Hz), 4.14 (2H, d, $J$ = 15.4 Hz), 4.45 (2H, d, $J$ = 15.4 Hz), 4.73 (1H, s), 6.69 (1H, d, $J$ = 2.3 Hz), 6.84 (1H, dd, $J$ = 2.3, 8.4 Hz), 6.92 (1H, t, $J$ = 7.3 Hz), 7.07 (2H, d, $J$ = 8.1 Hz), 7.32 (2H, t, $J$ = 7.8 Hz) and 7.39 (2H, d, $J$ = 8.4 Hz) ppm. LC/MS (ES$^+$) $t_r$ = 1.71 min (75 %), m/z 255.9 (M$^+$+H); (RP, Isocratic, 90% MeOH). HRMS (ES$^+$) calcd. $C_{16}H_{17}NNaO_2$ (M$^+$+Na) 278.1151, found 278.1153.

**2-(4-Clorophenyl)-7-methoxy-1,2,3,4-tetrahydroisoquinolin-4-ol (10g)**

The compound was synthesized according to method A. The crude compound was purified by column chromatography (eluent: from 0% to 40% EtOAc in pet. ether) to give a dark yellow wax (2.39 g, 31%) which showed: $^1$H NMR (500 MHz, CDCl$_3$) $\delta$ 2.36 (1H, d, $J$

= 8.6 Hz, OH), 3.32 (2H, dd, *J* = 2.5, 12.6 Hz, $H_3$-THIQ), 3.82 (3H, s, $OCH_3$), 3.82 (5H, ddd, *J* = 1.3, 3.5, 12.8 Hz, $H_3$-THIQ), 4.13 (2H, d, *J* = 15.3 Hz, $H_1$-THIQ), 4.42 (2H, d, *J* = 15.3 Hz, $H_1$-THIQ), 4.74 (1H, bs, $H_4$-THIQ), 6.69 (1H, d, *J* = 2.6 Hz, $H_8$-THIQ), 6.85 (1H, dd, *J* = 2.6, 8.5 Hz, $H_6$-THIQ), 6.98 (3H, d, *J* = 9.0 Hz, ArH, phenyl), 7.25 (4H, d, *J* = 9.0 Hz, ArH, phenyl) and 7.39 (1H, d, *J* = 8.5 Hz, $H_5$-THIQ) ppm. $^{13}$C NMR (126 MHz, $CDCl_3$) $\delta$ 51.3 ($C_1$-THIQ), 55.3 ($OCH_3$), 55.7 ($C_3$-THIQ), 66.7 ($C_4$-THIQ), 110.9 ($C_8$-THIQ), 113.4 ($C_6$-THIQ), 117.6 (ArCH, phenyl), 124.9 (ArCCl), 129.0 ($C_5CC_6$-THIQ), 129.1 (ArCH, phenyl), 130.5 ($C_5$-THIQ), 135.3 ($C_1CC_8$-THIQ), 149.6 (ArCN) and 159.4 ($C_7$-THIQ) ppm. LC/MS ($ES^+$) $t_r$ = 1.95 min (92 %), m/z 290.0 ($M^+$+H); (RP, Isocratic, 90% MeOH). HRMS ($ES^+$) calcd. for $C_{16}H_{17}{}^{35}ClNO_2$ ($M^+$+H) 290.0942, found 290.0935; calcd. $C_{16}H_{17}{}^{37}ClNO_2$ ($M^+$+H) 292.0913, found 292.0935.

**2-*p*-Tolyl-1,2,3,4-tetrahydroisoquinoline-4,6-diol (10i)**

The compound was synthesized according to method A. The crude compound was obtained as a brown-yellow solid (2.05 g) which showed: $^1$H NMR (400 MHz, $CDCl_3$) $\delta$ 3.31 (1H, dd, *J* = 2.5, 12.2 Hz), 3.76 (1H, dd, *J* = 3.5, 12.2 Hz), 4.07 (1H, d, *J* = 14.9 Hz), 4.36 (1H, d, *J* = 14.9 Hz), 4.69 (1H, s), 6.64 (1H, d, *J* = 8.7 Hz), 6.71 - 6.83 (2H, m) and 6.89 - 7.17 (4H, m) ppm. LC/MS ($ES^+$) $t_r$ = 1.52 min (70 %), m/z 256.1 ($M^+$+H); (RP, Isocratic, 90% MeOH).

**2-(4-methoxyphenyl)-1,2,3,4-tetrahydroisoquinoline-4,6-diol (10j)**

The compound was synthesized according to method A. The crude compound was obtained as a brown-yellow solid (2.17 g) which showed: $^1$H NMR (400 MHz, $CDCl_3$) $\delta$ 3.26 (1H, dd, *J* = 2.5, 12.4 Hz), 3.62 (1H, ddd, *J* = 1.1, 3.7, 12.2 Hz), 3.78 (3H, s), 4.00 (1H, d, *J* = 14.6 Hz), 4.25 (1H, d, *J* = 14.6 Hz), 4.66 (1H, t, *J* = 3.0 Hz), 6.77 (1H, dd, *J* = 2.7, 8.3 Hz), 6.87 (2H, d, *J* = 9.0 Hz), 6.90 (1H, d, *J* = 2.7 Hz), 6.99 (1H, d, *J* = 8.3 Hz) and

7.02 (2H, d, *J* = 9.0 Hz) ppm. LC/MS ($ES^+$) $t_r$ = 1.35 min (98 %), m/z 271.8 ($M^+$+H); (RP, Isocratic, 90% MeOH).

**2-(4-Chlorophenyl)-1,2,3,4-tetrahydroisoquinoline-4,6-diol (10k)**

The compound was synthesized according to method A. The crude compound was purified by column chromatography (eluent: 10% MeOH in DCM) to give a yellow-brown solid (1.88g, 95%) which showed: $^1$H NMR (500 MHz, $CDCl_3$) $\delta$ 2.63 (1H, bs, $C_4$OH), 3.37 (1H, dd, *J* = 2.2, 12.6 Hz, $H_3$-THIQ), 3.76 (1H, dd, *J* = 3.6, 12.6 Hz, $H_3$-THIQ), 4.11 (1H, d, *J* = 14.8 Hz, $H_1$-THIQ), 4.38 (1H, d, *J* = 14.8 Hz, $H_1$-THIQ), 4.72 (1H, s, $H_4$-THIQ), 5.30 (1H, bs, $C_6$OH), 6.81 (1H, dd, *J* = 2.3, 8.3 Hz, $H_7$-THIQ), 6.96 (1H, d, *J* = 2.2 Hz, $H_5$-THIQ), 6.99 (2H, d, *J* = 8.7 Hz, 2 x ArCH, phenyl), 7.05 (1H, d, *J* = 8.3 Hz, $H_7$-THIQ) and 7.27 (2H, d, *J* = 8.7 Hz, 2 x ArCH, phenyl) ppm. $^{13}$C NMR (126 MHz, $CDCl_3$) $\delta$ 50.8 ($C_1$-THIQ), 55.3 ($C_3$-THIQ), 67.1 ($C_4$-THIQ), 115.1 ($C_5$-THIQ), 115.9 ($C_7$-THIQ), 117.6 (2 x ArCH, phenyl), 125.0 (ArCCl), 125.9 ($C_1CC_8$-THIQ), 127.7 ($C_8$-THIQ), 129.1 (2 x ArCH, phenyl), 137.6 ($C_4CC_5$-THIQ), 149.6 (ArCN) and 154.6 ($C_6$-THIQ) ppm. LC/MS ($ES^+$) $t_r$ = 1.65 min (72 %), m/z 276.1 ($M^+$+H); (RP, Isocratic, 90% MeOH). HRMS ($ES^-$) calcd. for $C_{15}H_{13}ClNO_2$ ($M^-$-H) 274.0640, found 274.0629. Mp 168-171 °C

**6,7-Dimethoxy-2-(4-methoxyphenyl)-1,2,3,4-tetrahydroisoquinolin-4-ol (10l)**

The compound was synthesized according to method A. The crude compound was purified by column chromatography (eluent: from 0% to 50% EtOAc in pet. ether) to give the product as a white solid (3.54 g, 62%) which showed: $^1$H NMR (500 MHz, $CDCl_3$) $\delta$ 2.67 (1H, bs, OH), 3.23 (1H, dd, *J* = 2.3, 12.3 Hz, $H_3$-THIQ), 3.71 (1H, dd, *J* = 3.1, 12.3 Hz, $H_3$-THIQ), 3.79 (3H, s, $OCH_3$, phenyl), 3.88 (3H, s, $C_6OCH_3$-THIQ), 3.90 (3H, s, $C_7OCH_3$-THIQ), 4.01 (1H, d, *J* = 14.8 Hz, $H_1$-THIQ), 4.27 (1H, d, *J* = 14.8 Hz, $H_1$-THIQ), 4.67 (1H, bs, $H_4$-THIQ), 6.62 (1H, s, $H_8$-THIQ), 6.89 (2H, d, *J* = 9.0 Hz, 2 x ArCH, phenyl), 6.96 (1H,

s, $H_5$-THIQ) and 7.03 (2H, d, $J$ = 8.9 Hz, 2 x ArCH, phenyl) ppm. $^{13}$C NMR (126 MHz, $CDCl_3$) $\delta$ 52.7 ($C_1$-THIQ), 55.6 ($OCH_3$, phenyl), 55.9 (2 x $OCH_3$, THIQ), 57.2 ($C_3$-THIQ), 67.2 ($C_4$-THIQ), 108.6 ($C_8$-THIQ), 111.6 ($C_5$-THIQ), 114.5 (2 x ArCH, phenyl), 118.9 (2 x ArCH, phenyl), 126.8 ($C_1CC_8$-THIQ), 128.6 ($C_4CC_5$-THIQ), 145.3 (ArCN), 148.1 ($C_7$-THIQ), 148.9 ($C_6$-THIQ) and 154.2 (ArCO, phenyl) ppm. HRMS ($ES^+$) calcd. $C_{18}H_{22}NO_4$ ($M^+$+H) 316.1543, found 316.1543. Mp 136-137 °C (DCM/$Et_2O$).

**5,6,7-Trimethoxy-2-(4-methoxyphenyl)-1,2,3,4-tetrahydroisoquinolin-4-ol (10m)**

The compound was synthesized according to method A. The crude compound was purified by column chromatography (eluent: from 0% to 100% EtOAc in pet. ether) to give the product as a dark brown solid (5.49 g, 82%) which showed: $^{1}$H NMR (500 MHz, $CDCl_3$) $\delta$ 2.90 (1H, bs, OH), 3.18 (1H, dd, $J$ = 2.7, 12.4 Hz, $H_3$-THIQ), 3.70 (1H, dd, $J$ = 3.5, 12.6 Hz, $H_3$-THIQ), 3.79 (3H, s, $OCH_3$, phenyl), 3.86 (3H, s, $C_6OCH_3$-THIQ), 3.87 (3H, s, $C_7OCH_3$-THIQ), 3.98 (1H, d, $J$ = 15.1 Hz, $H_1$-THIQ), 4.02 (3H, s, $C_5OCH_3$-THIQ), 4.29 (1H, d, $J$ = 15.0 Hz, $H_1$-THIQ), 4.98 (1H, bs, $H_4$-THIQ), 6.45 (1H, s, $H_8$-THIQ), 6.88 (2H, d, $J$ = 9.0 Hz, 2 x ArCH, phenyl) and 7.04 (2H, d, $J$ = 9.0 Hz, 2 x ArCH, phenyl) ppm. $^{13}$C NMR (126 MHz, $CDCl_3$) $\delta$ 53.2 ($C_1$-THIQ), 55.6 ($OCH_3$, phenyl), 56.0 ($C_6OCH_3$-THIQ), 56.9 ($C_3$-THIQ), 60.9 ($C_7OCH_3$-THIQ), 61.5($C_5OCH_3$-THIQ), 62.7 ($C_4$-THIQ), 104.6 ($C_8$-THIQ), 114.5 (2 x ArCH, phenyl), 119.1 (2 x ArCH, phenyl), 123.0 ($C_1CC_8$-THIQ), 130.5 ($C_4CC_5$-THIQ), 140.6 ($C_7$-THIQ), 145.3 (ArCN), 152.2 ($C_5$-THIQ), 153.5 ($C_6$-THIQ) and 154.3 (ArCO, phenyl) ppm. HRMS ($ES^+$) calcd. $C_{19}H_{24}NO_5$ ($M^+$+H) 346.1649, found 346.1638.

**2-(3-Methoxyphenyl)-1,2,3,4-tetrahydroisoquinolin-4-ol (10o)**

The compound was synthesized according to method A. The crude compound was obtained as a yellow oil (1.73 g) which showed: $^{1}$H NMR (400 MHz, $CDCl_3$) $\delta$ 2.46 (1H, bs), 3.40 (1H, dd, $J$ = 2.7, 12.6 Hz), 3.83 (3H, s), 3.85 (1H, ddd, $J$ = 0.7, 3.9, 12.6 Hz), 4.21

(1H, d, $J$ = 15.5 Hz), 4.50 (1H, d, $J$ = 15.5 Hz), 4.79 (1H, s), 6.47 (1H, dd, $J$ = 2.3, 8.2 Hz), 6.61 (1H, t, $J$ = 2.3 Hz), 6.69 (1H, dd, $J$ = 2.3, 8.2 Hz), 7.17 - 7.20 (1H, m), 7.23 (1H, t, $J$ = 8.2 Hz), 7.28 - 7.32 (2H, m) and 7.45 - 7.53 (1H, m) ppm. LC/MS ($ES^+$) $t_r$ = 1.78 min (87 %), m/z 256.1 ($M^+$+H); (RP, Isocratic, 90% MeOH).

**1-(2-(Benzylamino)-4,6-dimethoxyphenyl)-2,2-dimethoxyethan-1-ol (11)**

The crude compound was purified by chromatography (eluent 0% to 40% EtOAc in pet. ether) to give the product as a pale yellow oil (1.94 g, 56%). $^1$H NMR (400 MHz, $CDCl_3$) $\delta$ 3.28 (3H, s, CHOC$\underline{H}_3$), 3.47 (3H, s, CHOC$\underline{H}_3$), 3.70 (3H, s, ArOC$\underline{H}_3$), 3.78 (3H, s, ArOC$\underline{H}_3$), 4.32 (2H, d, $J$ = 2.8 Hz, $ArCH_2$), 4.75 (1H, d, $J$ = 6.9 Hz, $CH(OR)_2$), 5.24 (1H, d, $J$ = 6.9 Hz, ArCH), 5.88 (1H, d, $J$ = 2.2 Hz, ArH), 5.94 (1H, d, $J$ = 2.2 Hz, ArH), 7.23 - 7.26 (1H, m, ArH) and 7.31 - 7.37 (4H, m, ArH) ppm. $^{13}$C NMR (101 MHz, $CDCl_3$) $\delta$ 48.0 ($ArCH_2$), 54.9 ($ArOCH_3$), 55.0 (CHO$\underline{C}H_3$), 55.7 ($ArOCH_3$), 55.9 (CHO$\underline{C}H_3$), 68.2 (ArCHOH), 88.2 (ArCH), 91.3 (ArCH), 103.7 (Ar$\underline{C}$CH), 105.2 ($CH(OR)_2$), 127.0 (ArCH), 127.3 (ArCH), 128.6 (ArCH), 139.7 (Ar$\underline{C}CH_2$), 149.4 (ArCN), 159.3 (Ar$\underline{C}OCH_3$) and 161.1 (Ar$\underline{C}OCH_3$) ppm. LC/MS (ES+) $t_r$ = 1.27 min (95%), m/z 348.0 ($M^+$+H); HRMS (ES+) calcd. for $C_{19}H_{26}NO_5$ ($M^+$+H) 348.1805, found 348.1793.

**5-Bromo-2-phenyl-1,2,3,4-tetrahydroisoquinolin-4-ol (14e)**

The compound was synthesized according to method A. The crude compound was obtained as a pale yellow oil (50 mg, 10%) which showed: $^1$H NMR (500 MHz, $CDCl_3$) $\delta$ 3.35 (1H, dd, $J$ = 2.7, 12.7 Hz, C$\underline{H}_2$CH), 3.77 (1H, ddd, $J$ = 1.0, 3.9, 12.7 Hz, C$\underline{H}_2$CH), 3.89 (1H, bs, OH), 4.13 (1H, d, $J$ = 15.6 Hz, $ArCH_2$), 4.37 (1H, d, $J$ = 15.6 Hz, $ArCH_2$), 4.72 (1H, t, $J$ = 3.3 Hz, C$\underline{H}$OH), 6.93 (1H, tt, $J$ = 0.9, 7.2 Hz, ArH), 7.03 (2H, dd, $J$ = 0.9, 8.7 Hz, ArH), 7.29 - 7.35 (4H, m, ArH) and 7.39 (1H, dd, $J$ = 2.0, 8.2 Hz, ArH) ppm. $^{13}$C NMR (126 MHz, $CDCl_3$) $\delta$ 51.0 ($ArCH_2$), 55.5 ($\underline{C}H_2$CH), 66.6 (CHOH), 116.7 (ArCH), 120.6 (ArCH),

121.9 (ArCBr), 129.3 (ArCH), 129.4 (ArCH), 130.3 (ArCH), 131.0 (ArCH), 135.7 (Ar<u>C</u>CH), 136.5 (Ar<u>C</u>$CH_2$) and 150.7 (ArCN) ppm. LC/MS ($ES^+$) $t_r$ = 2.20 min (86 %), m/z 303.4 ($M^+$+H) ($^{79}$Br), 305.4 ($M^+$+H) ($^{81}$Br); (RP, Isocratic, 90% MeOH). HRMS (ES+) calcd. for $C_{15}H_{15}{}^{79}BrNO$ ($M^+$+H) 304.0332, found 304.0321; calcd. for $C_{15}H_{15}{}^{81}BrNO$ ($M^+$+H) 306.0311, found 306.0320.

**1-Benzyl-4,5,6-trimethoxy-1*H*-indole (16p)**

The compound was synthesized according to method A. The crude compound was purified by chromatography (eluent from 0% to 30% EtOAc in pet. ether) to yield the product as a pale yellow oil (71 mg, 22%) which showed: $^1$H NMR (500 MHz, $CDCl_3$) $\delta$ 3.82 (3H, s, $CH_3$), 3.87 (3H, s, $CH_3$), 4.13 (3H, s, $CH_3$), 5.23 (2H, s, Ar$CH_2$), 6.47 (1H, s, ArH), 6.59 (1H, dd, $J$ = 0.8, 3.2 Hz, ArH), 6.94 - 6.97 (1H, m, ArH), 7.11 - 7.15 (2H, m, ArH) and 7.25 - 7.34 (3H, m, ArH) ppm. $^{13}$C NMR (126 MHz, $CDCl_3$) $\delta$ 50.2 (Ar$CH_2$), 56.3 ($CH_3$), 60.7 ($CH_3$), 61.4 ($CH_3$), 88.2 (ArCH), 99.2 (ArCH), 115.6 (Ar<u>C</u>CH), 126.2 (ArCH), 126.8 (ArCH), 127.6 (ArCH), 128.7 (ArCH), 133.4 (ArCN), 135.5 (Ar<u>C</u>O$CH_3$), 137.3 (Ar<u>C</u>$CH_2$), 145.9 (Ar<u>C</u>O$CH_3$) and 151.0 (Ar<u>C</u>O$CH_3$) ppm. LC/MS ($ES^+$) $t_r$ = 1.02 min (76 %), m/z 298.1 ($M^+$+H); (RP, Isocratic, 90% MeOH); HRMS (ES+) calcd. for $C_{18}H_{20}NO_3$ ($M^+$+H) 298.1438, found 298.1448.

***N*-Benzyl-2,2-dimethoxy-*N*-phenethylethanamine (18)**

2-Phenylethylamine (1.3 mL, 10.0 mmol) and benzaldehyde (1.0 mL, 10.0 mmol) were dissolved in $CHCl_3$ (50 mL) and treated with $NaBH(OAc)_3$ (3.3 g, 15.0 mmol). After stirring for 2 h at rt, 2,2-dimethoxyacetaldehyde (1.5 mL, 10.0 mmol) was introduced followed by $NaBH(OAc)_3$ (3.30 g, 15.0 mmol). After stirring for 6 h, the mixture was quenched with a saturated aqueous solution of $NaHCO_3$ and the aqueous layer was extracted with EtOAc (2 × 50 mL). The combined organics were dried with $MgSO_4$, filtered and evaporated to

give a pale green oil (2.52 g) which showed: $^{1}$H NMR (500 MHz, $CDCl_3$) δ 2.74 (2H, d, *J* = 5.2 Hz, CHC$\underline{H}_2$), 2.83 (4H, s, C$\underline{H}_2$C$\underline{H}_2$), 3.34 (6H, s, C$\underline{H}_3$), 3.77 (2H, s, Ar$CH_2$N), 4.43 (1H, t, *J* = 5.2 Hz, ArH), 7.15 - 7.23 (3H, m, ArH) and 7.24 - 7.37 (7H, m, ArH) ppm. $^{13}$C NMR (126 MHz, $CDCl_3$) δ 33.6 (Ar$\underline{C}H_2CH_2$), 53.9 ($CH_3$), 55.9 (CH$\underline{C}H_2$N), 56.7 ($CH_2\underline{C}H_2$N), 59.4 (Ar$CH_2$N), 104.2 ($CH(OR)_2$), 126.0 (ArCH), 127.0 (ArCH), 128.3 (ArCH), 128.4 (ArCH), 129.0 (ArCH), 129.0 (ArCH), 139.7 (Ar$\underline{C}CH_2$N) and 140.7 (Ar$\underline{C}CH_2CH_2$) ppm. LC/MS ($ES^+$) $t_r$ = 1.08 min (98 %), m/z 300.2 ($M^+$+H); (RP, Isocratic, 90% MeOH). HRMS (ES+) calcd. for $C_{19}H_{26}NO_2$ ($M^+$+H) 300.1958, found 300.1967.

**5,7,8,13-Tetrahydro-6,13-methanodibenzo[*c*,*f*]azonine (21)**

The compound was synthesized according to method A. The crude compound was obtained as a yellow oil (856 mg, 83%) which showed: $^{1}$H NMR (500 MHz, $CDCl_3$) δ 2.35 (1H, ddd, *J* = 1.3, 4.4, 15.7 Hz, ArC$\underline{H}_2CH_2$N), 3.10 (1H, ddd, *J* = 2.2, 12.9, 15.7 Hz, ArC$\underline{H}_2CH_2$N), 3.31 (1H, ddd, *J* = 2.2, 12.9, 15.0 Hz, Ar$CH_2$C$\underline{H}_2$N), 3.46 (1H, ddd, *J* = 1.3, 4.4, 15.0 Hz, Ar$CH_2$C$\underline{H}_2$N), 3.56 (1H, dd, *J* = 0.8, 13.9 Hz, NC$\underline{H}_2$CH), 3.67 (1H, ddd, *J* = 1.0, 5.2, 13.9 Hz, NC$\underline{H}_2$CH), 3.88 (1H, d, *J* = 5.2 Hz, $CH_2$C$\underline{H}$), 4.09 (1H, dd, *J* = 1.5, 17.1 Hz, Ar$CH_2$N), 4.61 (1H, d, *J* = 17.3 Hz, Ar$CH_2$N), 6.96 (1H, d, *J* = 7.4 Hz, ArH), 7.03 (1H, d, *J* = 8.6 Hz, ArH), 7.06 - 7.13 (3H, m, ArH), 7.13 - 7.22 (2H, m, ArH) and 7.32 (1H, dd, *J* = 1.2, 7.4 Hz, ArH) ppm. $^{13}$C NMR (126 MHz, $CDCl_3$) δ 34.7 (Ar$\underline{C}H_2CH_2$N), 44.5 ($CH_2\underline{C}$H), 52.5 (N$\underline{C}H_2$CH), 53.6 (Ar$CH_2$N), 56.2 (Ar$CH_2\underline{C}H_2$N), 125.1 (ArCH), 126.7 (ArCH), 126.8 (ArCH), 126.9 (ArCH), 126.9 (ArCH), 129.0 (ArCH), 130.4 (ArCH), 130.9 (ArCH), 134.6 (Ar$\underline{C}CH_2$N), 135.2 (Ar$\underline{C}$CH), 140.8 (Ar$\underline{C}CH_2CH_2$) and 145.0 (Ar$\underline{C}$CH) ppm. LC/MS ($ES^+$) $t_r$ = 0.95 min (99 %), m/z 236.0 ($M^+$+H); (RP, Isocratic, 90% MeOH). HRMS (ES+) calcd. for $C_{17}H_{18}N$ ($M^+$+H) 236.1434, found 236.1441.

**2,2-Dimethoxy-*N*-(3-methoxybenzyl)-*N*-(4-methoxybenzyl)ethanamine (22)**

*m*-Anisaldehyde (1.2 mL, 10.0 mmol) and 2,2-dimethoxyethylamine (1.1 mL, 10.0 mmol) were dissolved in $CHCl_3$ (50 mL) and treated with $NaBH(OAc)_3$ (3.3 g, 15.0 mmol). After stirring for 2 h at rt, *p*-anisaldehyde (1.2 mL, 10.0 mmol) was introduced followed by $NaBH(OAc)_3$ (3.30 g, 15.0 mmol). After stirring for 6h at rt, the mixture was quenched with a saturated aqueous solution of $NaHCO_3$ and the aqueous layer was extracted with EtOAc (2 × 50 mL). The combined organics were dried with $MgSO_4$, filtered and evaporated to give a pale green oil (3.49 g). The crude compound was purified by column chromatography to give the product as a colorless oil (2.78 g, 80%) which showed: 1H NMR (500 MHz, $CDCl_3$) δ 2.63 (2H, d, *J* = 5.2 Hz, $C\underline{H_2}CH$), 3.27 (6H, s, $CH(OC\underline{H_3})$), 3.56 - 3.66 (4H, m, $ArCH_2$), 3.80 (3H, s, $ArOCH_3$), 3.81 (3H, s, $ArOCH_3$), 4.46 (1H, t, *J* = 5.2 Hz, $CH(OR)_2$), 6.78 (1H, dd, *J* = 2.1, 7.8 Hz, ArH), 6.85 (2H, d, *J* = 8.7 Hz, ArH), 6.95 (2H, d, *J* = 7.8 Hz, ArH), 6.97 - 6.98 (1H, m, ArH), 7.22 (1H, t, *J* = 7.8 Hz, ArH) and 7.28 (2H, d, *J* = 8.7 Hz, ArH) ppm. $^{13}C$ NMR (126 MHz, $CDCl_3$) δ 53.6 $CH(O\underline{C}H_3)_2$, 55.0 ($\underline{C}H_2CH$), 55.3 ($ArOCH_3$), 55.4 ($ArOCH_3$), 58.5 ($ArCH_2$), 58.9 ($ArCH_2$), 104.0 (ArCH), 112.4 (ArCH), 113.7 (ArCH), 114.5 (ArCH), 121.3 (ArCH), 129.2 (ArCH), 130.2 (ArCH), 131.6 ($Ar\underline{C}CH_2$), 141.6 ($Ar\underline{C}CH_2$), 158.8 ($Ar\underline{C}OCH_3$) and 159.7 ($Ar\underline{C}OCH_3$) ppm. LC/MS ($ES^+$) $t_r$ = 1.03 min (96 %), m/z 346.3 ($M^+$+H); (RP, Isocratic, 90% MeOH). HRMS (ES+) calcd. for $C_{20}H_{28}NO_4$ ($M^+$+H) 346.2013, found 346.2025.

**7-Methoxy-2-(4-methoxybenzyl)-1,2,3,4-tetrahydroisoquinolin-4-ol (24)**

The compound was synthesized according to method A. The crude compound was purified by column chromatography (eluent: from 0% to 100% EtOAc in pet. ether) to give the product as a yellow oil (223 mg, 34%) which showed: $^1H$ NMR (500 MHz, $CDCl_3$) δ 2.61 (1H, dd, *J* = 2.6, 11.6 Hz $CHC\underline{H_2}$), 2.81 (1H, bs, OH), 3.05 (1H, ddd, *J* = 1.2, 3.1, 11.6 Hz, $CHC\underline{H_2}$), 3.33 (1H, d, *J* = 15.1 Hz, $ArC\underline{H_2}$ (THIQ)), 3.66 (2H, d, *J* = 5.1 Hz, $ArC\underline{H_2}$

(benzylic)), 3.76 (3H, s, $CH_3$), 3.76 (1H, d, *J* = 15.1 Hz, $ArCH_2$ (THIQ)), 3.81 (3H, s, $CH_3$), 4.56 (1H, bs), 6.53 (1H, d, *J* = 2.5 Hz, ArH (THIQ)), 6.79 (1H, dd, *J* = 2.5, 8.4 Hz, ArH (THIQ)), 6.88 (3H, d, *J* = 8.7 Hz, ArH (benzyl)), 7.28 (2H, d, *J* = 8.7 Hz, ArH (benzyl)) and 7.32 (1H, d, *J* = 8.4 Hz, ArH (THIQ)) ppm. $^{13}C$ NMR (126 MHz, $CDCl_3$) δ 55.4 ($CH_3$), 55.4 ($CH_3$), 56.0 ($ArCH_2$ (THIQ)), 58.5 ($CH\underline{C}H_2$), 62.1 ($ArCH_2$ (benzyl)), 66.9 ($\underline{C}HCH_2$), 110.8 (ArCH (THIQ)), 113.3 (ArCH (THIQ)), 113.9 (ArCH (benzyl)), 129.5 ($\underline{C}HCH_2$), 129.9 ($ArCH_2$ (benzyl)), 130.3 (ArCH (benzyl)), 130.7 (ArCH (THIQ)), 136.5 ($ArCH_2$ (THIQ)), 159.0 ($CH_3$) and 159.1 ($CH_3$) ppm. LC/MS ($ES^+$) $t_r$ = 0.88 min (69 %), m/z 300.0 ($M^+$+H); (RP, Isocratic, 90% MeOH). HRMS (ES+) calcd. for $C_{18}H_{22}NO_3$ ($M^+$+H) 300.1594, found 300.1587.

**2,9-Dimethoxy-7,12-dihydro-5H-6,12-methanodibenzo[c,f]azocine (25)**

The compound was synthesized according to method A. The crude compound was purified by column chromatography (eluent: from 0% to 30% EtOAc in pet. ether) to give the product as a brown gum (181 mg, 15%) which showed: $^1H$ NMR (500 MHz, $CDCl_3$) δ 3.35 (1H, d, *J* = 7.0 Hz, $CH\underline{H_2}$), 3.62 (1H, s, ArCH), 3.72 (3H, s, $CH_3$), 3.77 (3H, s, $CH_3$), 3.87 (1H, d, *J* = 14.0 Hz, $ArCH_2$), 3.90 (1H, d, *J* = 7.0 Hz, $CH\underline{H_2}$), 3.90 (1H, d, *J* = 14.5 Hz, $ArCH_2$), 4.52 (1H, d, *J* = 14.5 Hz, $ArCH_2$), 4.55 (1H, d, *J* = 14.0 Hz, $ArCH_2$), 6.52 (1H, d, *J* = 2.6 Hz, ArH), 6.63 (1H, dd, *J* = 2.6, 8.4 Hz, ArH), 6.66 (1H, dd, *J* = 2.6, 8.4 Hz, ArH), 6.75 (1H, d, *J* = 2.6 Hz, ArH), 6.89 (1H, d, *J* = 8.4 Hz, ArH) and 7.15 (1H, d, *J* = 8.4 Hz, ArH) ppm. $^{13}C$ NMR (126 MHz, $CDCl_3$) δ 35.9 ($\underline{C}HCH_2$), 49.4 ($CH\underline{C}H_2$), 55.2 ($CH_3$), 55.3 ($CH_3$), 56.9 ($ArCH_2$), 57.7 ($ArCH_2$), 110.8 (ArCH), 111.9 (ArCH), 112.4 (ArCH), 112.5 (ArCH), 126.0 ($Ar\underline{C}CH_2$), 127.1 (ArCH), 128.4 (ArCH), 132.8 ($Ar\underline{C}CH$), 135.5 ($Ar\underline{C}CH_2$), 142.1 ($Ar\underline{C}CH$), 157.8 ($Ar\underline{C}OCH_3$) and 157.9 ($Ar\underline{C}OCH_3$) ppm. LC/MS ($ES^+$) $t_r$ = 0.90 min (94 %), m/z 282.2 ($M^+$+H); (RP, Isocratic, 90% MeOH). HRMS (ES+) calcd. for $C_{18}H_{20}NO_2$ ($M^+$+H) 282.1489, found 282.1482.
